# A novel Time-resolved Fluoroimmunoassay for the quantitative detection of Antibodies against the Phospholipase A2 Receptor

**DOI:** 10.1038/srep46096

**Published:** 2017-04-11

**Authors:** Biao Huang, Liang Wang, Yi Zhang, Jue Zhang, Qiuhua Zhang, Hualong Xiao, Bin Zhou, Zhuxing Sun, Ya-nan Cao, Yu Chen, Zhigang Hu, Huiming Sheng

**Affiliations:** 1 Jiangsu Institute of Nuclear Medicine, Wuxi, 214063, China; 2 Wuxi People’s Hospital affiliated to Nanjing Medical University, Wuxi, 214023, China; 3 Tongren Hospital,Shanghai Jiao Tong University School of Medicine, Shanghai, 200336, China; 4 The First Affiliated Hospital, College of Medicine, Zhejiang University, Hangzhou, 310003, China

## Abstract

A highly sensitive time-resolved fluoroimmunoassay (TRFIA) was developed to quantify serum antibodies against the phospholipase A2 receptor (anti-PLA2R-IgG) for differential diagnosis of membranous nephropathy. Recombinant PLA2R (rPLA2R) was coated onto 96-well plates as a capture. A goat-anti-human IgG tracer was prepared with europium-chelate for detection. After bound/free separation by washing, the fluorescence counts of bound tracer were measured for quantifying serum anti-PLA2R-IgG concentration. A purified anti-PLA2R-IgG calibrator was first prepared for ensuring that consistent quantitative results could be obtained. The assay detection limit was 0.03 mg/L with linear measurement range of 0.03–340 mg/L. The intra- and inter-assay coefficients of variation (CVs) were 3.8% and 6.2%, respectively. The average serum anti-PLA2R-IgG concentration in 45 healthy volunteers, 31 IgA nephropathy, 9 lupus nephropathy, and 52 idiopathic membranous nephropathy patients was 0.53 ± 0.18 mg/L, 0.70 ± 0.41 mg/L, 1.08 ± 0.65 mg/L, and 9.00 ± 11.82 mg/L, respectively. The cut-off point for an abnormal anti-PLA2R-IgG concentration was defined as >0.89 mg/L. The positive rates in serum from patients with IgA nephropathy, lupus nephropathy, and idiopathic membranous nephropathy were 29.0%, 44.4%, and 88.5%, respectively. The availability of this quantitation method will facilitate the use of serum anti-PLA2R-IgG for diagnosing idiopathic membranous nephropathy.

Nephritic syndrome (NS) is a kidney disease characterized by increased glomerular basement membrane permeability, and typically presents with symptoms of nephritic range proteinuria, hypoalbuminemia, severe edema, and hyperlipidemia. Membranous nephropathy (MN) is a common cause of NS in adults. Two different forms of MN have been described based on its pathogenesis: primary idiopathic membranous nephropathy (iMN), which is found in 80% of MN patients, and a secondary form associated with various malignancies, autoimmune diseases, and some infections^1^,^2^,^3^. MN often has an asymptomatic onset, and ~ 20% of patients show no proteinuria in the nephritic range. Although a conventional physical examination can identify some patients with proteinuria, the infeasibility of performing an invasive renal biopsy for all suspected cases of MN results in its delayed diagnosis. MN displays a slow progression, and some patients enter a spontaneous remission phase; however, 30~40% of patients eventually develop end-stage renal disease or die^4^,^5^,^6^.

MN is associated with deposits of immune complex in the subepithelial and intramembranous regions of tissue. In 2009, the target antigen of membranous nephropathy, phospholipase A2 receptor (PLA2R), was detected on the surface of normal cells found in the serum of 70% of iMN patients^7^. Here, we employed a highly sensitive time-resolved fluoroimmunoassay (TRFIA), to quantitatively detect anti-PLA2R antibodies in serum (Fig. 1), and then used the method to detect anti-PLA2R antibodies in the serum of kidney disease patients.

## Results

**Figure 2:**
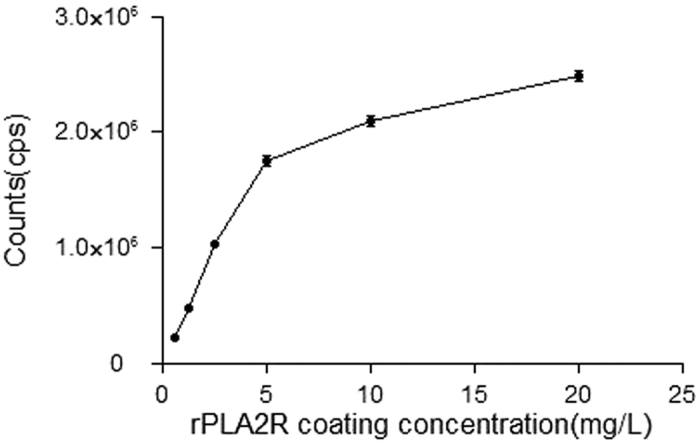
Binding curves at different rPLA2R coating concentrations.

**Figure 3:**
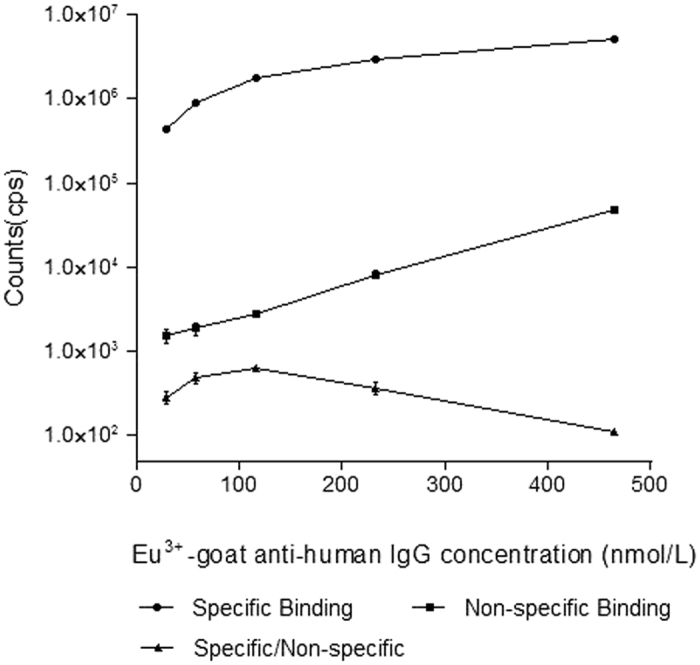
Specific and non-specific binding curves at different Eu^3+^-goat anti-human IgG concentrations.

**Figure 4:**
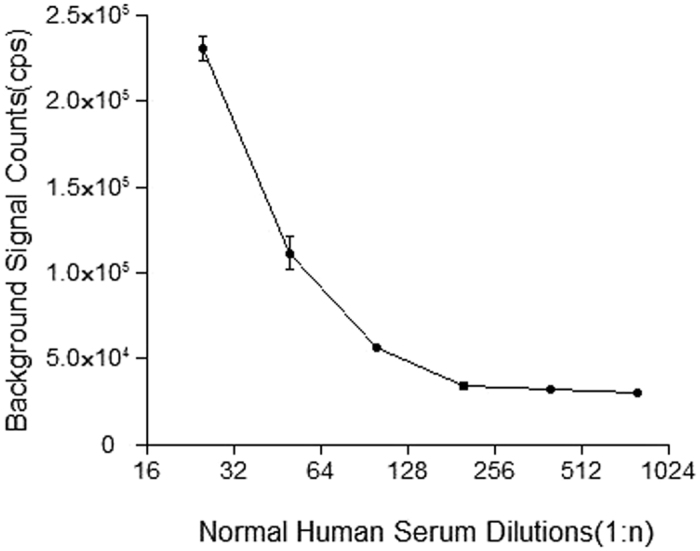
Non-specific binding curves at different dilutions of normal human serum.

**Figure 5:**
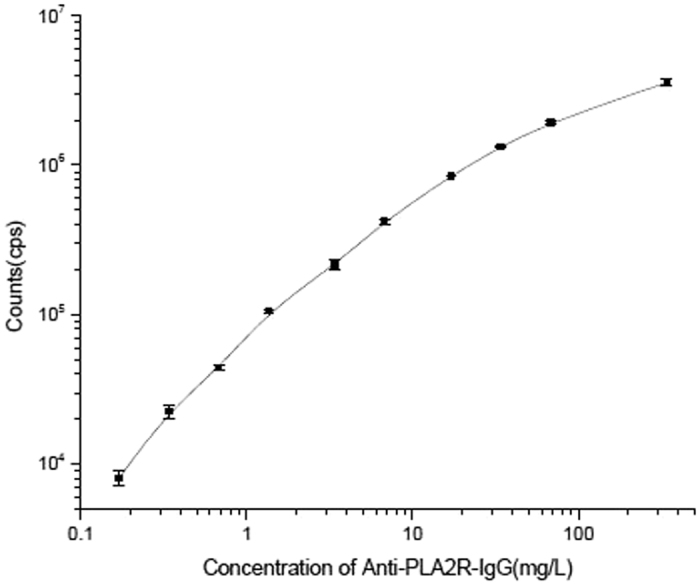
Calibration curve for the anti-PLA2R-IgG TRFIA.

Each parameter of the TRFIA for detection of anti-PLA2R IgG in human serum was optimized to ensure the assay’s reliability. The rPLA2Rs were diluted with coating buffer to concentrations of 0.625 mg/L–20 mg/L, and coated overnight at 4 °C. After blocking, a high concentration of anti-PLA2R IgG was added to each well which was coated with a different concentration of the rPLA2R (3 wells per concentration); after which, the corresponding fluorescence counts were detected. The optimal coating concentration was determined to be ~5 mg/L (Fig. 2), as the counts per second (cps) readouts became nearly saturated at concentrations >5 mg/L. When optimizing the Eu^3+^-goat anti-human IgG concentration, the background remained low at low concentrations. Moreover, the specific binding shown by a high concentration rPLA2R standard was also low (Fig. 3). When a high concentration of the Eu^3+^-labeled antibody was used, the background increased, which may affect the assay’s detection sensitivity. The specific to non-specific binding ratio was highest when the Eu^3+^ -goat anti-human IgG concentration was 116 nmol/L; therefore, we deemed that concentration to be optimal. Due to the large number of IgG molecules in serum, non-specific binding was difficult to avoid, thus the serum sample was diluted prior to detection. The samples of normal human serum were diluted to ratios of 1:25, 1:50, 1:100, 1:200, 1:400, and 1:800, respectively, prior to analysis. As the fluorescence counts did not significantly change at dilutions >1:200, that dilution was designated as optimal (Fig. 4). Finally, a calibration curve was obtained by using different concentrations of the calibrator (Fig. 5), and the detection limits of the assay were determined. The assay’s sensitivity, as defined by the concentration of anti-PLA2R-IgG which corresponded to the fluorescence of the zero calibrators (+3 standard deviations), was 0.03 mg/L. The working range of the anti-PLA2R-IgG TRFIA was 0.03–340 mg/L, and its EC50 was 57.2 mg/L. The assay had a linear response range of 0.17–34 mg/L, and within that range, the correlation coefficient was 0.992.

When different concentrations of an anti-PLA2R standard were added, the recovery rates ranged from 91.7–103.2%, and the mean recovery rate was 98.2%. When the quality-controlled samples were assayed at three different concentrations, the intra- and inter-batch coefficients of variation (CV) were 3.8% and 6.2%, respectively.

**Figure 6:**
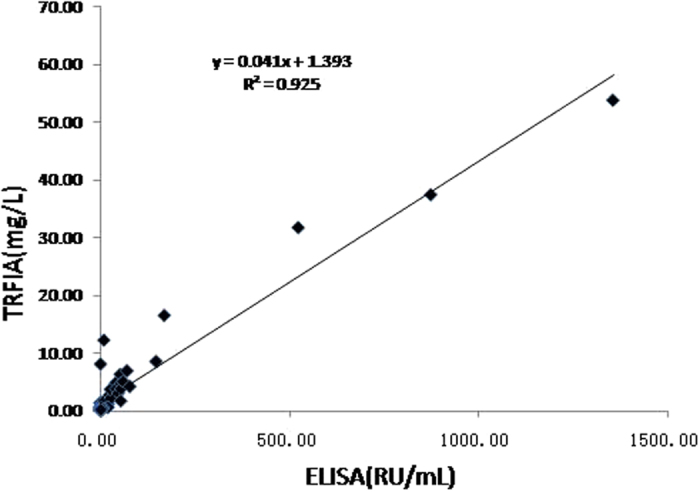
Linear correlation between the anti-PLA2R-IgGTRFIA and ELISA.

A total of 59 serum samples were assayed using our TRFIA and a serum anti-PLA2R-IgG-ELISA manufactured by EUROIMMUN Inc. (Luebeck, Germany); the results are shown in Fig. 6. The inter-assay correlation coefficient of the TRFIA and ELISA was 0.925, demonstrating that results obtained with the TRFIA were consistent with those obtained with the ELISA. However, the sensitivity and working range of the TRFIA were better than those of the ELISA, as there were 9 iMN patients who tested positive with the TRFIA but negative with the ELISA (Table 1).

**Figure 7:**
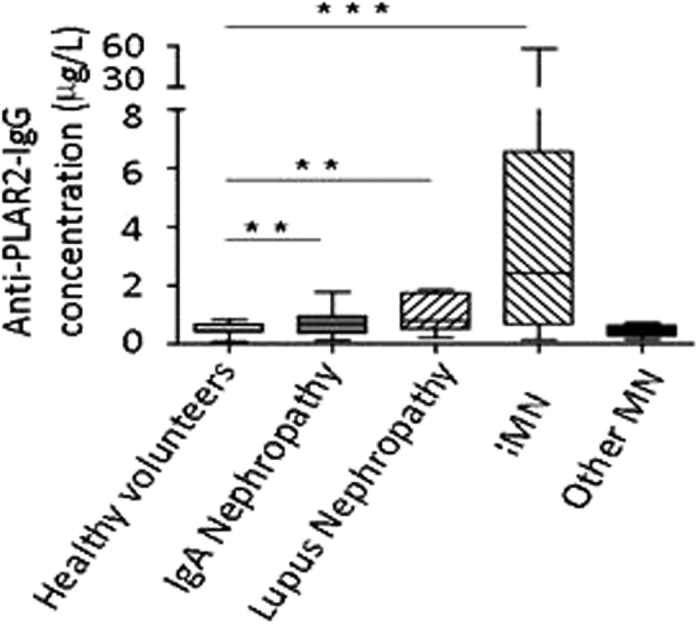
Anti-PLA2R-IgG levels in the serum of patients with the indicated nephropathies.

The serum samples used for assay development were divided into 5 groups: 45 controls, 52 cases of iMN, 31 cases of IgA nephropathy, 9 cases of lupus nephropathy, and 9 cases of other kidney diseases (2 cases were Henoch-Schonlein purpura nephritis, 3 cases were diabetic nephropathy, and 4 cases were hepatitis B virus-associated glomerulonephritis). The concentration of PLA2R antibodies in each serum sample was detected in duplicate using the TRFIA method, and the results are shown in Fig. 7. The ability to determine serum anti-PLA2R-IgG concentrations can play an important role in the accurate diagnosis of kidney disease, as those relative concentrations are high in cases of iMN, intermediate in cases of lupus erythematosus nephropathy, and low in cases of IgA nephropathy.

The mean anti-PLA2R-IgG concentration in serum obtained from healthy control subjects was 0.53 mg/L, and the normal range, calculated as the mean ± 2SDs, was 0.17–0.89 mg/L. The cut-off point for a normal anti-PLA2R level was set at <0.89 mg/L. The rates of anti-PLA2R-IgG detection in cases of iMN, IgA nephropathy, and lupus nephropathy were 88.5%, 29.0%, and 44.4%, respectively (Table 2).

## Discussion

A diagnosis of membranous nephropathy mainly relies on a patient’s clinical features and the pathological changes observed in a renal biopsy specimen. While biopsy results are the gold criteria for diagnosis, this method has some obvious disadvantages, as it can produce postoperative trauma, secondary damage to the kidney that might aggravate an existing illness, or result in a puncture wound infection caused by the spread of bacteria from the original kidney infection. During the years in which MN is treated, renal biopsy is a painful, risky, and expensive approach for assessing disease progression. Thus, many patients refuse biopsy, and thereby sacrifice their opportunity for early diagnosis and treatment. Therefore, it is important to develop new sensitive and accurate methods for diagnosing MN, and a serological test would be a major breakthrough for patients with this disease.

Anti-PLA2R-IgG is an appropriate serological marker for iMN. Because PLA2R is heavily glycosylated, treatment with peptide N-glycosidase F causes a shift in its electrophoretic mobility to approximately 145 kd^8^. Furthermore, anti-PLA2R can accurately identify the 185-kD PLA2R glycoprotein extracted from the non-reducing glomerulus as well as the rPLA2R^9^. Antibodies against PLA2R are found in the majority iMN patients, signifying that PLA2R is a major antigen associated with the disease. Moreover, it has been suggested that serum levels of PLA2R autoantibodies can be used for diagnosing iMN and monitoring a patient’s response to treatment^7^,^9^,^10^,^11^,^12^.

Anti-PLA2R-IgG in serum is an ideal biomarker for diagnosing MN and evaluating possible curative treatments. While western blot methods have previously detected PLA2R autoantibodies only in iMN patients and not in patients with other kidney diseases^7^, this autoantibody has recently been detected in some secondary MNs such as type V lupus nephritis, hepatitis B virus (HBV)-related MN, and tumor-related MN^9^. Thus, a sensitive test for detecting anti-PLA2R-IgG is critical for distinguishing among the various types of MN. Because the titers of PLA2R autoantibody change throughout iMN treatment^13^, it is necessary to perform a quantitative analysis of the autoantibodies. While westernblotting has been used in previous studies, that method mainly produces a qualitative or semi-quantitative analysis, because it has limited sensitivity. Furthermore, western blotting requires the use of native purified glomerular glycoproteins or extracts obtained from cells that overexpress PLA2R, as well as electrophoresis and blotting apparatus this is not available in all clinical laboratories. Several immunofluorescence cell-based assays (IIFCBA) and ELISAs based on recombinant human PLA2R have been developed to detect and quantify antibodies against PLA2R^4^,^14^,^15^,^16^,^17^,^18^,^19^,^20^. Hoxha *et al*.^15^ described an indirect immunofluorescence test (IIFT) that enabled the easy and specific detection of antibodies against PLA2R in serum. When using that IIFT, antibodies against PLA2R were found in 52% of patients with biopsy-proven iMN, but in no patients with secondary MN^15^. Debiec^10^ reported that antibodies against PLA2R were not observed in the sera of some iMN patients; however, the PLA2R antigen was found in their immune deposits^10^. The PLA2R antibody positive rates reported for iMN patients varied from 52% to 81%, and the fluctuation in positive rates reported for patients with secondary MN was even wider. Some possible explanations for these inconsistent results include: (1) different methods, often using a color depth or absorbance concentrations, were used to assess the concentrations of antibodies against PLA2R; (2) low assay sensitivity may have made it difficult to distinguish between different kidney diseases. The observation that some patients with iMN did not have antibodies against PLA2R could be explained by limitations of the current immunoassays. The time-resolved fluorescence immunoassay (TRFIA) offers certain advantages, such as its good reproducibility and quantification characteristics, being easy to automate, not requiring the use of radioactive isotopes, and the high stability of its reagents. The TRFIA provides greatly increased sensitivity because it relies on labeling with a non-radioactive atom rather the use of a macromolecule enzyme; this permits higher degrees of labeling to be obtained. Furthermore, the use of atom labeling reduces the assay’s effect on biological activity, increases methodological stability with the large Stoke shift between the excitation and emission wavelengths, and effectively avoids environmental interference, because the fluorescence emission cycle is relatively long. Addition of a fluorescence enhancement solution during the detection phase of the assay can enhance the original fluorescence by 1 million-fold; thus the detection limit of the TRFIA is 10^−18^ mol/L, which is considerably higher than the 10^−10^ mol/L limit of ELISA. All these features greatly improve the assay’s range and sensitivity. To verify that our assay could be used for a quantitative analysis, anti-PLA2R-IgG molecules were extracted, purified, and prepared as quantitative calibrators, which could be used to help ensure consistent results. The sensitivity of the anti-PLA2R-IgG TRFIA is 0.03 mg/L, which allows it to distinguish 88.5% of iMN cases, which is superior to the capability of other research methods. Previous studies reported that a small percentage of patients (5–25%) with secondary MN are positive for serum anti–PLA2R-IgG as detected by westernblotting, the IIF-CBA, and ELISA^9^,^21^,^22^. If true, this could disturb the differential diagnosis of iMN. In our research, the anti-PLA2R-IgG levels in patients with IgA nephropathy, lupus nephropathy, and other types of MN were either slightly elevated or ≤1.85 mg/L, which would still be significantly different from the levels in patients with iMN.

In conclusion, the anti-PLA2R-IgG TRFIA has a high degree of sensitivity, and can be helpful for diagnosing and classifying cases of iMN.

## Materials and Methods

### Chemicals and instrumentation

Human IgG and goat anti-human IgG antibodies were obtained from Jackson ImmunoResearch (West Grove, PA, USA). The europium-labeling kit (1244–302), including N_1_-(p-isothiocyanatobenzyl)-diethylenetriamine-N_1_,N_2_,N_3_,N_4_-tetraacetic acid (DTTA), was purchased from Perkin Elmer (Waltham, MA, USA). Diethylenetriaminepentaacetate (DTPA), bovine serum albumin (BSA), Tris, and Triton X-100 were purchased from Sigma (St. Louis, MO, USA). A Sephadex-G50 column was purchased from Pharmacia (England), and CentriconYM-50 ultrafiltration tubes were purchased from Millipore (Billerica, MA, USA). Ninety-six-well polystyrene microtiter plates were purchased from Nunc International (Denmark). β-Naphthoyltrifluoroacetone (β-NTA) was synthesized in our laboratory. Purified water was produced using a Barnstead water purification system. All other reagents were of analytical grade and purchased from domestic manufacturers. A model DU-650 spectrometer from Beckman (Germany) was used to detect proteins at 280 nm when collecting antibodies during the purification process. An _Auto_DELFIA_1235_ automatic immunoassay system from Perkin Elmer was used to measure Eu^3+^ fluorescence in microtiter wells. A serum anti-PLA2R-IgG-ELISA kit was purchased from EUROIMMUN Inc (Germany).

### Blood samples

Samples of blood serum were obtained from 45 healthy volunteers who showed no evidence of nephropathy, a gastroduodenal disorder, or liver disease at Jiangyuan Hospital (China). The study was approved by the institutional research ethics committee of Jiangsu Institute of Nuclear Medicine. All enrolled subjects provided their written informed consent for study participation, and all methods were performed in accordance with the relevant guidelines and regulations. Serum samples were also collected from 101 kidney disease patients at the Affiliated Wuxi People’s Hospital of Nanjing Medical University (China). Each patient had undergone a renal biopsy and a pathological examination at the time of blood collection. All methods were performed in accordance with the relevant guidelines and regulations. Among the 101 patients, 52 were iMN, 31 were IgA nephropathy, 9 were lupus erythematosus nephropathy, 2 were Henoch-Schonlein purpura nephritis, 3 were diabetic nephropathy, and 4 were hepatitis B virus-associated glomerulonephritis. iMN was diagnosed by means of a renal biopsy in patients who lacked features suggesting secondary membranous nephropathy. The study protocol was approved by the Institutional Review Board of the Affiliated Wuxi People’s Hospital of Nanjing Medical University, all enrolled patients provided their written informed consent for study participation, and all methods were performed in accordance with the relevant guidelines and regulations.

### Expression and purification of rPLA2R

rPLA2R was generated by cloning into 293 T cells. Briefly, the 293 T cells were seeded into culture plates (cell surface = 10 cm) at a density of 70–80% confluence, and incubated for 24 hours prior to transfection. Next, 25 μL of Mega Tran 1.0 transfection reagent and 5 μg of plasmids were transferred into 500 μL of serum-free medium and incubated for 20 min. The mixture was then added to the cell cultures, with shaking. After 48 hours of transfection, the cells were harvested and lysed on ice with lysis buffer for 15 min; after which, the lysate was collected.

rPLA2R was purified on a Sephadex column packed with 0.3 mL of column material that was coupled with anti-Flag antibody. The column was first washed with 5 volumes of TBST, and then another 3 times with one volume of glycine solution (0.1 M, pH 3.5). Next, the column was washed 3 times with 10 volumes of TBST and 2 times with ten volumes of lysis buffer. The column was then loaded 3 times with 40 mL of cell lysate that had been filtered through a 0.45 μm membrane filter; after which, the column was washed once with 10 volumes of lysis buffer, three times with 10 volumes TBST, and then eluted with 500 uL of glycine solution (0.1 M, pH 3.5). The eluant was adjusted to pH 7.3 with 1 M Tris buffer (pH 9.0), and 10% glycerin was added for storage.

### Development of the anti-PLA2R-IgG TRFIA for use with clinical serum samples

#### Preparation of the capture antigen and secondary antibody

Coating buffer was prepared by diluting the coating antigen (rPLA2R) to the desired concentration with 50 mmol/L Na_2_CO_3_-NaHCO_3_ (pH 9.6). Next, a 100 μL aliquot of the coating buffer was placed into individual wells of a 96-well microtiter plate, which was then incubated overnight at 4 °C. Following incubation, each well was washed three times, and the plate was then blocked by addition of 200 μL of blocking solution (50 mmol/L Na_2_CO_3_-NaHCO_3_, pH 9.6, containing 3 g/L BSA) to each well, followed by an overnight incubation at 4 °C. After incubation, the blocking buffer was discarded, each well was vacuum dried, and the plate was tightly sealed and stored at −20 °C. Goat-anti-human IgG was diluted with 50 mmol/L Na_2_CO_3_-NaHCO_3_ (pH 9.6) buffer to a final concentration of 10 mg/L. The diluted antibody was then added to the 96-well microtiter plates, (100 μL per well), and the plates were incubated and processed as for rPLA2R.

#### Preparation of the Eu^3+^-goat anti-human IgG antibody

A 1 mL aliquot of goat anti-human IgG antibody (2 g/L) was dissolved in 50 mmol/L PBS (pH 7.0) and loaded onto a PD-10 column for buffer conversion. The elution buffer at 50 mmol/L Na_2_CO_3_-NaHCO_3_ (pH 9.0) contained 155 mmol/L NaCl. The protein peak was collected and quantitatively analyzed for its UV absorption. A 500 μL volume of diluted goat anti-human IgG antibody was added to a vial containing 0.2 mg of Eu^3+^-N_2_-[p-isocyanate-benzyl]-DTTA, and the resulting reaction was facilitated by magnetic stirring for 16 h at 28–30 °C. The reaction solution was then injected onto a Sephadex-G50 column (1 × 40 cm) that had been equilibrated with 80 mmol/L Tris-HCl (pH 7.8). The fractions from the first peak containing the highest Eu^3+^ fluorescence counts were pooled and characterized. The labelled goat anti-human IgG was diluted with elution buffer that contained 0.2% BSA as a stabilizer, and stored at –20 °C until future use.

#### Preparation of human anti-PLA2R IgG calibrators

To date, no quantitative standard has existed for anti-PLA2R-IgG. However, a standard was required to ensure that each quantitative experiment would be comparable. We prepared such a standard via the following steps. 1) Extraction of human anti-PLA2R IgG: The serum from an iMN patient was diluted 1:200 with analysis buffer, and then pipetted into 6 wells (100 μL per well) that were coated with rPLA2R. Two additional wells received 100 μL of analysis buffer as non-specific adsorption (NSB) solution. Next, the plates were placed on a shaker for 2 hours at 37 °C, and then washed three times with washing solution. Next, 100 μL of 6 M urea was added to each of three wells, and the plates were shaken for 20 min at 37 °C. The specific IgG dissolved in urea solution was then removed for quantitative IgG detection. The three wells were washed twice with washing liquid, and antibodies against PLA2R were detected in the other 5 wells to determine whether any antibody remained in a non-dissociated state. 2) Quantitative detection of specific IgG: The urea solution containinganti-PLA2R IgG, taken from the above 3 wells, was ultrafiltered with a YM-50 Ultrafiltration tube, and then transferred into buffer (50 mM Tris-HCl, pH 7.8, containing 8 mM NaCl) at a final volume of 120 μL. The concentration of anti-PLA2R IgG in buffer was determined by using a standard curve created for human IgG as follows: 50 μL of human IgG standard of different concentrations and 50 μL of human specific IgG against PLA2R were pipetted into individual wells coated with goat anti-human IgG. Next, 50 μL of assay buffer was added, and the plates were shaken for 2 hours at 37 °C; after which, the wells were washed three times with washing buffer. Next, 100 μL of Eu^3+^ goat anti-human IgG antibody diluted in assay buffer was pipetted into each well, and the plates were shaken for 1 hour at 37 °C. Following this incubation, the plates were washed 6 times with washing buffer. Finally, 200 μL of enhancement solution was added, and the fluorescence counts were detected after 5 min of shaking. The concentration of a specific IgG was calculated using the standard curve created for human IgG. A sample with a known specific IgG concentration was diluted into serial concentrations that served as standards for antibodies against PLA2R.

### Reagent preparation

The luminescent enhancement system used a solution containing 2-naphthoyltrifluoroacetone (β-NTA). One liter of enhancement solution contained 15 μmoles of β-NTA, 50 μmoles of tri-n-octylphosphine oxide, and 1 mL of Triton X-100, pH 3.2. The assay buffer contained 50 mmol/L Tris-HCl (pH 7.8), 8 mmol/L NaCl, 0.1% BSA, 50 μmol/L DTPA, 100 mmol/L Tween-80, and 0.1% NaN_3_.

### Anti-PLA2R-IgG detection procedure

A 100 μL volume anti-PLA2R-IgG standard or a sample (diluted 1:200 in assay buffer) was pipetted into each well of a rPLA2R-coated microtiter plate, which was then incubated for 1 h at 25 °C with agitation. After 3 times rinses, Eu^3+^-goat anti-human IgG antibody diluted in assay buffer was added (100 μL/well), and the plate was incubated for 1 h at 25 °C with agitation. The plate was then washed 6 times; after which, enhancement solution was added (200 μL/well). The plate was agitated for 5 minutes before any measurements were taken. All procedures were auto-controlled by the autoDELFIA_1235_ immunoassay system that was equipped with software designed in our laboratory. A calibration curve was created, and the concentrations of target molecules in unknown samples were automatically calculated using a Multicalc software program, which used a spline algorithm for analyzing logarithmically transformed data.

### Assessment of the anti-PLA2R-IgG TRFIA method

#### Sensitivity

Mean values (X) and standard deviations (SD) were calculated for the count values at the zero concentration points on the standard curves for each of the 10 groups. Each assay’s sensitivity was calculated as the concentration corresponding to the value of  X + 3 SD, as determined via the standard curve.

#### Recovery rate

After determining the TRFIA’s background response produced by nonspecific binding, three standard concentrations of anti-PLA2R-IgG were added to the samples, and the ratio of each measured value to its theoretical value was calculated.

#### Precision

The intra- and inter-batch coefficients of variation (CV) were determined from measurements obtained by analyzing quality-controlled samples prepared at three different concentrations.

## Additional Information

**How to cite this article:** Huang, B. *et al*. A novel Time-resolved Fluoroimmunoassay for the quantitative detection of Antibodies against the Phospholipase A2 Receptor. *Sci. Rep.* **7**, 46096; doi: 10.1038/srep46096 (2017).

**Publisher's note:** Springer Nature remains neutral with regard to jurisdictional claims in published maps and institutional affiliations.

## Figures and Tables

**Figure 1:**
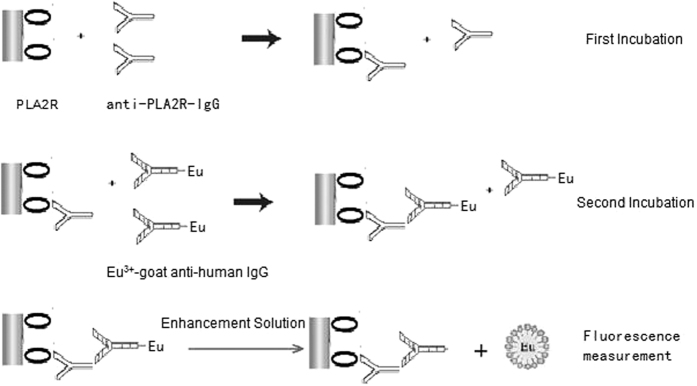
A schematic representation of the anti-PLA2R-IgG-TRFIA.

**Table 1:** Comparison of the TRFIA and the ELISA

	Healthy volunteers (n = 20)	iMN (n = 39)
TRFIA positive rates	0/20 (0%)	35/39 (89.7%)
ELISA positive rates	0/20 (0%)	26/39 (66.7%)

**Table 2:** The concentrations of serum anti-PLA2R-IgG in different nephropathy diseases.

	Healthy volunteers (n = 45)	IgA nephropathy (n = 31)	lupus nephropathy (n = 9)	other kidney disease n = 9	iMN (n = 52)
mean ± SD (mg/L)	0.53 ± 0.18	0.70 ± 0.41	1.08 ± 0.65	0.42 ± 0.20	9.00 ± 11.82
positive rates	0	29.0%	44.4%	0	88.5%
